# Association Between Familial Mediterranean Fever and P-Wave Dispersion Under Colchicine Treatment

**DOI:** 10.3390/diagnostics16091252

**Published:** 2026-04-22

**Authors:** Osman Cüre, Hüseyin Durak, Mustafa Çetin, Bayram Kızılkaya

**Affiliations:** 1 Department of Rheumatology, Faculty of Medicine, Recep Tayyip Erdoğan University, 53020 Rize, Türkiye; 2 Department of Cardiology, Faculty of Medicine, Recep Tayyip Erdoğan University, 53020 Rize, Türkiye; huseyin.durak@erdogan.edu.tr (H.D.); mustafa.cetin@erdogan.edu.tr (M.Ç.); 3 Department of Internal Medicine, Training and Research Hospital, Recep Tayyip Erdoğan University, 53020 Rize, Türkiye; bayram.kizilkaya@saglik.gov.tr

**Keywords:** Familial Mediterranean Fever, P-wave dispersion, colchicine, chronic inflammation

## Abstract

**Background/Objectives:** The relationship between P-wave dispersion (Pd) and disease status in patients with Familial Mediterranean Fever (FMF) undergoing colchicine treatment is unclear in the literature, and results are contradictory. This study aimed to evaluate P-wave dispersion in patients with Familial Mediterranean Fever receiving regular long-term colchicine treatment and to compare these findings with those of age- and sex-matched individuals without FMF. **Methods:** A cross-sectional and observational study included 97 individuals with positive FMF and 97 individuals with negative FMF. FMF diagnosis was confirmed according to the Tel-HaShomer criteria, and all patients received regular colchicine treatment and were evaluated during the attack-free period. P maximum, P minimum, and Pd were measured using standard 12-lead electrocardiography (ECG); clinical, laboratory, and drug data were recorded. Pd associations were analyzed using correlation and multivariable regression. **Results:** Pd was found to be significantly higher in FMF (+) patients (47 vs. 39 ms, *p* < 0.001). Pd showed a positive correlation with FMF status (r = 0.508, *p* < 0.001), colchicine dose (r = 0.476, *p* < 0.001), white blood cell (WBC) (r = 0.209, *p* = 0.005) and high-density lipoprotein cholesterol (HDL-C) (r = 0.156, *p* = 0.037) and a negative correlation with calcium channel blocker use (r = −0.245, *p* = 0.001). In multivariate analysis, FMF increased Pd by 10.17 ms, while calcium channel blockers decreased it by 11.78 ms (*p* < 0.001). Age, WBC and HDL-C also had independent positive effects on Pd (*p* < 0.001, *p* = 0.017, *p* = 0.040, respectively). **Conclusions:** These findings suggest that FMF is associated with increased P-wave dispersion despite regular colchicine treatment, indicating persistent subclinical atrial conduction heterogeneity.

## 1. Introduction

Familial Mediterranean Fever (FMF) is a hereditary autoinflammatory disorder characterized by recurrent episodes of fever and serosal inflammation, including peritonitis, pleuritis, arthritis, and erysipelas-like skin lesions. FMF is caused by mutations in the *MEFV* gene, which encodes pyrin, a key regulator of inflammatory pathways [1,2]. Recent advances in FMF pathogenesis and genotype–phenotype correlations have improved understanding of disease variability and personalized management [3,4,5,6]. Disease severity varies according to *MEFV* mutations; for example, M694V is associated with more severe disease, whereas E148Q is generally milder [4,5]. Databases such as INFEVERS catalog numerous *MEFV* variants and support studies exploring their impact on clinical heterogeneity and treatment response [6].

Chronic subclinical inflammation in FMF may persist between attacks, potentially affecting the cardiovascular system even in the absence of overt symptoms [7,8,9]. Among cardiac manifestations, atrial conduction abnormalities are of particular interest, as their prevalence in FMF is not well established, and clinical significance remains uncertain. Even if clinically apparent atrial arrhythmias are uncommon, subclinical conduction changes detectable by P-wave dispersion (Pd) may indicate early atrial remodeling and predispose to future supraventricular arrhythmias and may provide clinically relevant early markers of atrial involvement, supporting the importance of investigating Pd even in the absence of overt arrhythmias [10,11,12].

P-wave dispersion on electrocardiography, defined as the difference between the maximum and minimum P-wave durations, reflects atrial conduction heterogeneity and is associated with an increased risk of supraventricular arrhythmias such as atrial fibrillation [10,11,12]. Although clinically significant atrial arrhythmias are relatively uncommon in FMF, subclinical conduction heterogeneity detectable by Pd may reveal the impact of persistent inflammation on atrial electrophysiology and provide early markers of cardiovascular involvement.

Previous studies investigating Pd in FMF patients have reported conflicting results, with some showing increased Pd and others finding no significant difference [13,14,15]. Furthermore, while colchicine is known to suppress inflammation and may reduce cardiovascular risk, its effect on atrial conduction remains uncertain [16].

The aim of this study was to evaluate P-wave dispersion in patients with Familial Mediterranean Fever receiving regular long-term colchicine treatment and to compare these findings with those of age- and sex-matched individuals without FMF.

## 2. Materials and Methods

### 2.1. Study Design

This study was designed as a consultation-based cross-sectional and observational study consisting of age- and gender-matched 97 FMF (+) and 97 FMF (−) individuals. This study was approved by the Ethics Committee of Recep Tayyip Erdoğan University Faculty of Medicine (Date: 4 December 2025, No: 2025/486). All data were collected after ethical approval between December 2025 and January 2026. This research followed internationally accepted ethical guidelines as outlined in the Declaration of Helsinki, and all participants gave written informed consent.

### 2.2. Patient Selection and Clinical-Laboratory Evaluation

FMF diagnosis was made in patients to be included in the cohort using Tel-HaShomer clinical criteria. Tel-HaShomer criteria include major/minor findings such as recurrent typical inflammatory attacks, monoarticular arthritis, serositis attacks and positive response to treatment, and FMF is diagnosed with the presence of 1 major or 2 minor criteria together [3]. FMF (−) group included age- and sex-matched individuals who did not meet the Tel-HaShomer criteria and had no clinical history of FMF. All participants were selected from the hospital population, and none received colchicine. Baseline characteristics, comorbidities, medication use, laboratory values, and electrocardiographic parameters are summarized in Table 1. All participants included in this study were of Turkish origin.

Inclusion criteria: Individuals in the FMF (+) group meeting Tel-HaShomer criteria, healthy individuals without FMF diagnosis in the matched control group, and patients receiving colchicine treatment at 1 mg/day or higher for one year or longer. Exclusion criteria: Significant structural heart disease, severe electrolyte disturbances or conditions where medications affect electrocardiographic parameters, and those using antiarrhythmic drugs for any arrhythmia.

### 2.3. Matching Process

Matching based on age and gender was performed using the nearest neighbor method. After matching, a total of 194 individuals were analyzed, with 97 patients in the FMF group and 97 in the other group. Matching aimed to minimize potential confounding effects.

### 2.4. Data Collection and Evaluation

Detailed questionnaire-based demographic data (age, gender), clinical characteristics (smoking, body mass index, comorbidities), and treatment information were obtained from the hospital’s electronic medical record system. In all individuals diagnosed with FMF, the diagnosis was confirmed according to the Tel-HaShomer criteria, and only patients receiving colchicine treatment and in the attack-free period were included in the analysis. The attack-free period was defined as the absence of typical FMF attack symptoms (fever, serositis, arthritis, or erysipelas-like erythema) for at least two weeks prior to enrollment, without evidence of an acute inflammatory episode at the time of evaluation. This approach was used to minimize the potential transient effects of acute inflammatory attacks on electrocardiographic parameters, particularly P-wave dispersion. The colchicine dose was recorded in mg/day.

Laboratory data were obtained from blood samples taken as close to the time of electrocardiographic evaluation as possible and after at least 8 h of fasting. Hematological parameters (white blood cell count, neutrophil count, hemoglobin), biochemical parameters (fasting glucose, creatinine, glomerular filtration rate), and C-reactive protein (CRP) levels as an inflammatory marker were recorded. Total cholesterol (TC), low-density lipoprotein cholesterol (LDL-C), and high-density lipoprotein cholesterol (HDL-C) levels were analyzed as part of the lipid profile. All laboratory measurements were performed in the hospital’s accredited central laboratory using standard automated analyzers.

Treatment-related variables included colchicine dosage along with the use of calcium channel blockers, beta-blockers, angiotensin-converting enzyme (ACE) inhibitors/angiotensin receptor blockers (ARB), and statins. These drugs, known to affect electrocardiographic parameters, were considered potential confounding factors and included in the statistical analysis model. The aim was to more accurately determine the independent effect of FMF status on P-wave dispersion.

### 2.5. Electrocardiography

Standard 12-lead ECG recordings were obtained at a paper speed of 25 mm/s and calibration of 10 mm/mV after at least 10 min of rest in the supine position. P-wave measurements were manually performed on magnified digital ECG images by two independent cardiologists who were blinded to all clinical and laboratory data.

To assess measurement reproducibility and minimize observer-related bias, 30 ECG recordings were randomly selected and re-evaluated. Each observer performed measurements independently without access to the other observer’s results. For intra-observer variability, the same observer repeated the measurements of the selected ECG recordings at a different time point under similar conditions, without reference to the initial measurements.

Inter-observer and intra-observer variability were assessed using intraclass correlation coefficient (ICC) analysis. The results demonstrated good inter-observer agreement and excellent intra-observer reproducibility for P-wave dispersion measurements, with an inter-observer ICC of 0.82 and an intra-observer ICC of 0.91.

P-wave maximum (Pmax) and minimum (Pmin) durations were determined by identifying the longest and shortest P-wave durations across all leads. The onset of the P wave was defined as the first upward or downward deflection from the isoelectric line, and the offset was defined as the point of return to the isoelectric line. Measurements were performed in the lead where the P wave was most clearly visualized.

P-wave dispersion (Pd) was calculated as the difference between Pmax and Pmin and expressed in milliseconds [17]. A methodological quality and risk-of-bias assessment was performed using the revised Joanna Briggs Institute (JBI) critical appraisal tool for analytical cross-sectional studies, and the completed appraisal is provided in Supplementary Materials [18].

### 2.6. Statistical Methods

Statistical analyses were performed using SPSS software (version 29; IBM Corp., Armonk, NY, USA). Continuous variables were assessed for normal distribution using the Shapiro–Wilk test. Normally distributed variables were presented as mean ± standard deviation, whereas non-normally distributed variables were expressed as median (interquartile range). Continuous variables were compared using the independent samples *t*-test or Mann–Whitney U test, as appropriate. Categorical variables were compared using the chi-square test or Fisher’s exact test. Correlation analyses were performed using Pearson or Spearman correlation coefficients, depending on data distribution. Multivariable linear regression analyses were conducted to identify independent predictors of P-wave dispersion. Variables with potential clinical relevance were included in the regression models. A backward stepwise approach was used in additional analyses to confirm the robustness of the results. A two-sided *p* value < 0.05 was considered statistically significant.

HDL-C was included in the regression model because of its well-established anti-inflammatory, antioxidant, and endothelial-protective properties. In patients with familial Mediterranean fever (FMF), chronic subclinical inflammation and endothelial dysfunction are important components of disease pathophysiology. HDL-C has been shown to modulate inflammatory pathways and oxidative stress, which may influence atrial electrical remodeling. Furthermore, reduced HDL-C levels have been associated with impaired endothelial function and increased cardiovascular risk in inflammatory conditions. Given that atrial conduction abnormalities reflected by increased P-wave dispersion may be influenced by systemic inflammation and endothelial dysfunction, HDL-C was considered a biologically plausible covariate in the regression models.

A post hoc power analysis was performed to evaluate the statistical power of the study to detect the observed effect size for the association between FMF and P-wave dispersion. Based on the final regression model and the total sample size of 194 participants, the statistical power of the study was estimated to be approximately 0.92 at a significance level of 0.05, indicating that the study was adequately powered to detect the observed associations.

## 3. Results

In the age- and sex-matched cohort, the FMF (+) (*n* = 97) and FMF (−) (*n* = 97) groups were similar in terms of demographic characteristics (age: 36.35 ± 12.39 vs. 37.94 ± 12.42 years, *p* = 0.42; female proportion: 41.2% in both groups, *p* = 1.00). Body mass index (BMI), smoking, diabetes, and coronary artery disease prevalence did not differ significantly between the groups. Cardiovascular comorbidities such as coronary artery disease (1.0% vs. 6.2%, *p* = 0.120) and diabetes mellitus (4.1% vs. 11.3%, *p* = 0.110) were numerically lower in FMF (+) patients but did not reach statistical significance. However, hypertension (6.2% vs. 16.5%, *p* = 0.027) and hyperlipidemia (26.8% vs. 44.3%, *p* = 0.006) were significantly less prevalent in FMF (+) patients. Similarly, the use of cardiovascular medications was lower in the FMF (+) group, including ACE inhibitors or ARBs (4.1% vs. 15.5%, *p* = 0.016), beta-blockers (0% vs. 24.7%, *p* < 0.001), and calcium channel blockers (4.1% vs. 17.5%, *p* = 0.006). Statin use was infrequent in both groups (0% vs. 4.1%, *p* = 0.130). As expected, all FMF (+) patients were receiving colchicine (1 mg/day or higher) while none of the FMF (−) participants received colchicine (*p* < 0.001). Laboratory parameters including white blood cell count (7.12 [5.84–8.22] vs. 6.68 [5.78–8.02] × 10^3^/µL, *p* = 0.780) and neutrophil count (4.33 [3.27–5.49] vs. 3.86 [3.14–5.25] × 10^3^/µL, *p* = 0.160) were numerically higher in FMF (+) patients, but differences were not statistically significant. No significant differences were observed in hemoglobin, glucose, creatinine, eGFR, or lipid profile components such as total cholesterol, HDL-C, and LDL-C. However, CRP, an inflammatory marker, was significantly higher in FMF (+) patients (5.00 [2.00–10.00] vs. 3.10 [1.91–5.85] mg/L, *p* = 0.019), reflecting ongoing subclinical inflammation despite colchicine therapy. Electrocardiographic parameters revealed that FMF (+) patients had significantly higher P maximum (108.00 [104.00–116.00] vs. 106.00 [100.00–114.00] ms, *p* = 0.012) and P-wave dispersion (47.00 [42.00–51.00] vs. 39.00 [31.00–43.00] ms, *p* < 0.001), whereas P minimum was slightly lower (64.00 [57.00–69.00] vs. 65.00 [59.00–77.00] ms, *p* = 0.022). These findings suggest increased atrial conduction heterogeneity in FMF patients (Table 1).

**Figure 1:**
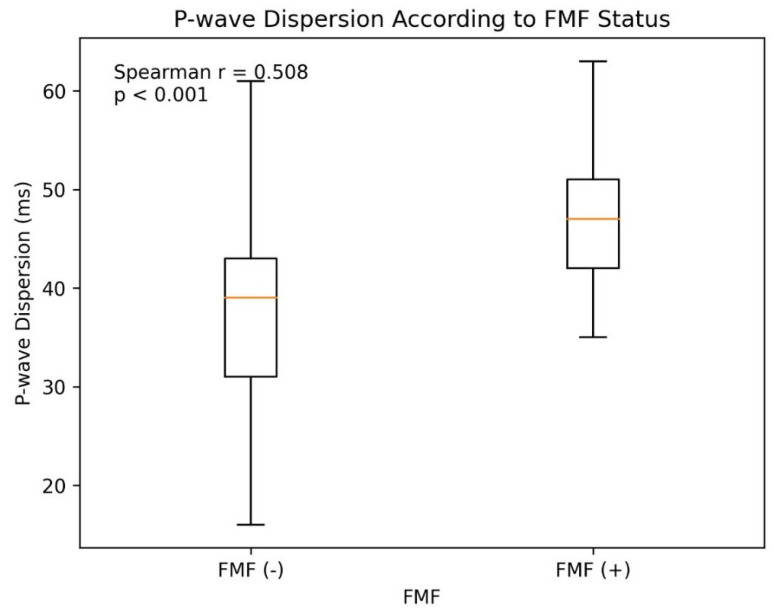
P-wave dispersion according to FMF status.

P-wave dispersion was significantly higher in FMF (+) individuals compared to FMF (−) individuals (Spearman r = 0.508, *p* < 0.001). The distribution of P-wave dispersion according to FMF status is shown in Figure 1. Correlation analyses (Table 2) showed that P-wave dispersion was strongly associated with FMF status (r = 0.508, *p* < 0.001) and colchicine dose (r = 0.476, *p* < 0.001). Among laboratory markers, white blood cell count (r = 0.209, *p* = 0.005), neutrophil count (r = 0.198, *p* = 0.007), CRP (r = 0.173, *p* = 0.021), and HDL-C (r = 0.156, *p* = 0.037) were positively correlated with P-wave dispersion. Calcium channel blocker use was inversely correlated (r = −0.245, *p* = 0.001), while other clinical variables such as age, sex, BMI, hypertension, diabetes, hyperlipidemia, and coronary artery disease showed no significant correlation.

To address potential multicollinearity between white blood cell (WBC) and neutrophil counts, two separate multivariable backward linear regression analyses were performed. In the analysis including WBC (Table 3A), FMF status was associated with an average increase of 10.37 ms in P-wave dispersion (95% CI: 8.05–12.70, *p* < 0.001). WBC count also showed a significant positive association with P-wave dispersion (β = 0.79 ms per 10^3^/µL increase, 95% CI: 0.20–1.37, *p* = 0.009). Age (β = 0.26 ms/year, 95% CI: 0.15–0.37, *p* < 0.001) and HDL-C (β = 0.11 ms per mg/dL, 95% CI: 0.01–0.21, *p* = 0.028) were positively associated with P-wave dispersion, whereas CCB use was associated with a reduction in P-wave dispersion (β = −12.35 ms, 95% CI: −17.71 to −6.98, *p* < 0.001). This analysis explained 31.3% of the variance in P-wave dispersion (Adjusted R^2^ = 0.313). In the analysis including neutrophil count instead of WBC (Table 3B), FMF status (β = 10.21 ms, 95% CI: 7.85–12.57, *p* < 0.001), age (β = 0.27 ms/year, 95% CI: 0.16–0.38, *p* < 0.001), HDL-C (β = 0.13 ms per mg/dL, 95% CI: 0.03–0.23, *p* = 0.015), and CCB use (β = −12.76 ms, 95% CI: −18.21 to −7.31, *p* < 0.001) remained significant predictors of P-wave dispersion. The variance explained in this analysis was slightly lower, at 29.6% (Adjusted R^2^ = 0.296). These analyses confirm the robustness of our findings and the independent association of FMF with increased P-wave dispersion, even after accounting for inflammatory markers, age, HDL-C, and CCB use.

**Figure 2:**
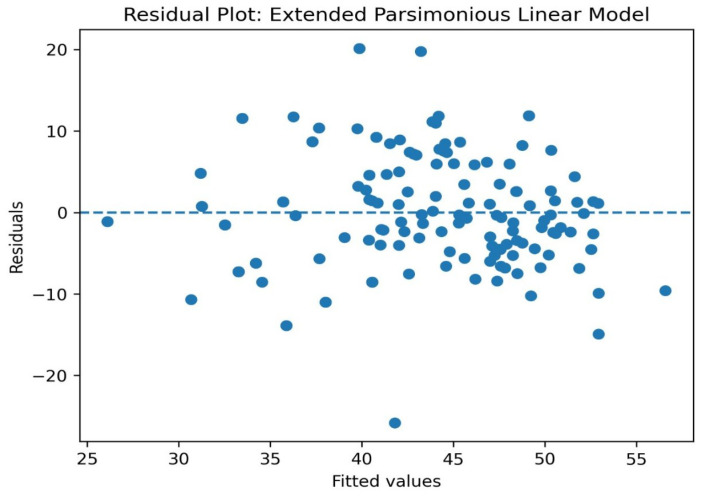
Residual plot: Extend Parsimonious Linear Model.

Residual plots revealed that the residuals were symmetrically distributed around zero and did not show a distinct pattern with fitted values (Figure 2).

## 4. Discussion

In this study, electrocardiographic evaluations of age- and sex-matched Familial Mediterranean Fever (FMF) (+) and FMF (−) cohorts revealed significantly higher P wave dispersion (Pd) in the FMF group. This finding suggests that the inflammatory process of FMF may increase atrial conduction heterogeneity. In chronic inflammatory conditions such as FMF, systemic inflammation has been shown to lead to long-term vascular and cardiac electrophysiological changes; FMF patients have been reported to have a higher prevalence of cardiovascular pathologies such as ischemic heart disease, atrial fibrillation, stroke, and heart failure [14,19,20]. These findings support the relationship between inflammation and cardiovascular risk.

Studies have shown that chronic inflammation in FMF has adverse effects on the cardiovascular system even at the subclinical level; in particular, vascular changes such as arterial stiffness and endothelial dysfunction have been demonstrated in FMF, and this may create a basis for cardiac conduction heterogeneity [21,22].

However, the literature on Pd in FMF is inconsistent. While some reports found no significant difference in P wave dispersion and other conduction parameters in FMF, some studies reported significant changes in ventricular repolarization indices. In particular, while the positive effects of colchicine treatment on ventricular repolarization parameters (e.g., Tp E, Tp E/QT) were highlighted, no significant change was reported in P wave dispersion [23]. This suggests that although the anti-inflammatory effect of colchicine is reflected in ventricular repolarization indices, its effect on Pd, a measure of atrial conduction heterogeneity, may be more limited or variable. Importantly, our study design does not allow the independent effect of colchicine to be determined, since all FMF-positive patients were receiving colchicine while none of the controls were exposed to this treatment. Therefore, the observed association between colchicine dose and P-wave dispersion should not be interpreted as a direct causal or harmful electrophysiological effect of colchicine. In clinical practice, higher colchicine doses are typically prescribed in patients with more severe inflammatory phenotypes; thus, colchicine dose likely reflects residual inflammatory burden rather than a direct determinant of atrial conduction heterogeneity.

Although our study did not include direct *MEFV* genotyping, evidence from previous FMF cohorts supports the relevance of genotype–phenotype associations in disease expression and severity. A recent study comparing Italian and Lebanese FMF patients reported that Lebanese individuals had more frequent and severe clinical manifestations, associated with a higher prevalence of pathogenic variants such as M694V, whereas Italians had milder phenotypes with different variant distributions [24]. Similarly, genotype–phenotype studies have shown that certain mutations (e.g., M694V and V726A) are linked to more severe phenotypes and higher attack frequencies compared to other variants [25,26]. These observations suggest that genetic background may partly explain the clinical heterogeneity seen in FMF and might contribute to differences in electrophysiological parameters such as P-wave dispersion, highlighting the importance of future studies incorporating genetic analyses.

The correlations found between inflammation markers (e.g., CRP or long-term effects of inflammation) and Pd in our study support the idea that chronic inflammation may affect electrophysiological parameters. It has been previously suggested that subclinical inflammation persists even outside of inflammatory attacks in FMF, leading to vascular changes [27,28,29].

Our study also found a negative correlation between calcium channel blocker use and Pd. In chronic inflammatory conditions such as FMF, calcium channel blockers can modulate P wave dispersion by reducing atrial conduction heterogeneity. This finding suggests that pharmacological agents may have potential interactions on electrophysiological parameters and that these mechanisms of action should be investigated in detail [30,31].

**Limitations:** The study focused solely on electrocardiographic P-wave dispersion and did not include broader arrhythmia assessments such as advanced cardiac imaging and Holter monitoring. Therefore, although increased P-wave dispersion may indicate atrial conduction heterogeneity, its direct relationship with future atrial arrhythmia risk could not be determined in this study.

Because all FMF-positive participants were receiving colchicine and none of the controls were using colchicine, the independent effect of colchicine on P-wave dispersion could not be determined in this study.

We acknowledge that CCB use was recorded as a binary variable (use vs. non-use), and information regarding specific CCB subclasses (dihydropyridine vs. non-dihydropyridine) was not available. Therefore, we could not perform stratified analyses according to CCB subclasses. Future studies with detailed medication data may clarify potential differential effects of CCB subclasses on P-wave dispersion.

The lack of assessment of *MEFV* genotype and detailed FMF-related disease severity parameters may limit interpretation of the findings, since different FMF genotypes and inflammatory phenotypes may influence atrial conduction properties. In addition, attack frequency, disease duration, serial inflammatory markers, and colchicine adherence were not consistently available due to the retrospective design. These factors may affect inflammatory burden and electrophysiological findings and should be addressed in future prospective studies.

Additionally, all participants were recruited from the same hospital in Turkey, so environmental and lifestyle factors were likely similar across groups. However, differences in such factors in other populations may affect the generalizability of our findings. Generalizability may be limited as data were collected from a single center.

## 5. Conclusions

This study demonstrates that P-wave dispersion is higher in FMF patients receiving colchicine compared with matched controls, suggesting persistent subclinical atrial conduction heterogeneity despite long-term colchicine treatment. However, because direct arrhythmia monitoring was not performed, these findings should be interpreted as subclinical electrophysiological observations rather than direct predictors of atrial fibrillation risk. Larger prospective studies integrating rhythm monitoring, inflammatory burden, and genotype data are required.

## Figures and Tables

**Table 1:** Comparison of Demographic, Clinical, Laboratory, and Electrocardiographic Characteristics Between FMF-Positive and FMF-Negative Groups Matched for Age and Sex.

Variable	FMF (−) (n = 97)	FMF (+) (n = 97)	*p* Value
Age (years)	37.94 ± 12.42	36.35 ± 12.39	0.420
Sex, male, n (%)	40 (41.2%)	40 (41.2%)	1.000
Smoking, n (%)	20 (20.6%)	12 (12.4%)	0.130
BMI (kg/m^2^)	26.07 ± 4.32	25.62 ± 4.31	0.490
CAD, n (%)	6 (6.2%)	1 (1.0%)	0.120
DM, n (%)	11 (11.3%)	4 (4.1%)	0.110
HT, n (%)	16 (16.5%)	6 (6.2%)	0.027
HPL, n (%)	43 (44.3%)	26 (26.8%)	0.006
ACE inh./ARB use, n (%)	15 (15.5%)	4 (4.1%)	0.016
Beta-blocker use, n (%)	24 (24.7%)	0 (0.0%)	<0.001
CCB use, n (%)	17 (17.5%)	4 (4.1%)	0.006
Statin use, n (%)	4 (4.1%)	0 (0.0%)	0.130
Colchicine dose (mg/day)	0 (0.0%)	97 (100%)	<0.001
WBC (10^3^/µL)	6.68 [5.78–8.02]	7.12 [5.84–8.22]	0.780
Neutrophil (10^3^/µL)	3.86 [3.14–5.25]	4.33 [3.27–5.49]	0.160
Hgb (g/dL)	13.30 [12.60–15.40]	13.00 [12.00–15.00]	0.190
Glucose (mg/dL)	92.00 [85.00–100.00]	91.00 [84.00–96.00]	0.200
eGFR (mL/dk/1.73 m^2^)	111.00 [95.00–123.00]	112.00 [100.00–122.50]	0.510
Creatinine (mg/dL)	0.68 [0.60–0.86]	0.70 [0.60–0.90]	0.720
Total cholesterol (mg/dL)	187.00 [144.00–245.00]	184.00 [166.75–209.00]	0.380
HDL-C (mg/dL)	46.40 [40.20–50.00]	46.50 [40.00–55.00]	0.510
LDL-C (mg/dL)	116.00 [85.00–158.00]	112.00 [95.00–132.25]	0.210
CRP (mg/L)	3.10 [1.91–5.85]	5.00 [2.00–10.00]	0.019
P max (ms)	106.00 [100.00–114.00]	108.00 [104.00–116.00]	0.012
P min (ms)	65.00 [59.00–77.00]	64.00 [57.00–69.00]	0.022
P-wave dispersion (ms)	39.00 [31.00–43.00]	47.00 [42.00–51.00]	<0.001

FMF, Familial Mediterranean Fever; BMI, body mass index; CAD, coronary artery disease; DM, diabetes mellitus; HT, hypertension; HPL, hyperlipidemia; ACE inh.; angiotensin-converting enzyme inhibitors; ARB, angiotensin receptor blocker; CCB, calcium channel blocker; WBC, white blood cell; Hgb, hemoglobin; eGFR, estimated glomerular filtration rate; HDL-C, high-density lipoprotein cholesterol; LDL, low-density lipoprotein; CRP, C-reactive protein.

**Table 2:** Correlation of P-wave Dispersion with Clinical, Laboratory and Treatment Variables.

Variable	r	*p* Value
Age (years)	0.129	0.073
Sex	0.117	0.105
Smoking	−0.160	0.033
BMI (kg/m^2^)	0.063	0.401
FMF status	0.508	<0.001
CAD	−0.079	0.285
DM	0.042	0.569
HT	−0.131	0.073
HPL	0.118	0.107
ACE inh./ARB use	−0.063	0.383
Beta-blocker use	−0.089	0.219
CCB use	−0.245	0.001
Statin use	−0.081	0.261
Colchicine dose (mg/day)	0.476	<0.001
WBC (10^3^/µL)	0.209	0.005
Neutrophil (10^3^/µL)	0.198	0.007
Hgb (g/dL)	−0.089	0.234
Glucose (mg/dL)	−0.144	0.052
eGFR (mL/min/1.73 m^2^)	−0.090	0.228
Creatinine (mg/dL)	0.057	0.441
TC (mg/dL)	0.006	0.933
HDL-C (mg/dL)	0.156	0.037
LDL-C (mg/dL)	−0.098	0.190
CRP (mg/L)	0.173	0.021

FMF, Familial Mediterranean Fever; BMI, body mass index; CAD, coronary artery disease; DM, diabetes mellitus; HT, hypertension; HPL, hyperlipidemia; ACE inh.; angiotensin-converting enzyme inhibitors; ARB, angiotensin receptor blocker; CCB, calcium channel blocker; WBC, white blood cell; Hgb, hemoglobin; eGFR, estimated glomerular filtration rate; TC, total cholesterol; HDL-C, high-density lipoprotein cholesterol; LDL-C, low-density lipoprotein cholesterol; CRP, C-reactive protein. Correlation coefficients (r) with *p*-values are shown. *p* < 0.05 indicates statistical significance.

**Table 3:** (**A**). Multivariable backward linear regression analysis of P-wave dispersion including white blood cell count, FMF, Age, HDL-C, and Calcium Channel Blocker Use (Adjusted R^2^ = 0.313). (**B**) Multivariable Backward Linear Regression Analysis of P-wave Dispersion Including FMF, Age, HDL-C, and Calcium Channel Blocker Use (Adjusted R^2^ = 0.296).

(A)			
Variable	β Coefficient	95% CI	*p* Value
FMF	10.37	8.05–12.70	<0.001
WBC	0.79	0.20–1.37	0.009
Age	0.26	0.15–0.37	<0.001
HDL-C	0.11	0.01–0.21	0.028
CCB	−12.35	−17.71–−6.98	<0.001
**(B)**			
**Variable**	**β Coefficient**	**95% CI**	***p* Value**
FMF	10.21	7.85–12.57	<0.001
Age	0.27	0.16–0.38	<0.001
HDL-C	0.13	0.03–0.23	0.015
CCB	−12.76	−18.21–−7.31	<0.001

FMF, Familial Mediterranean Fever; WBC, white blood cell count; HDL-C, high-density lipoprotein cholesterol; CCB, calcium channel blocker. *p* < 0.05 indicates statistical significance.

## Data Availability

The original contributions generated for this study are included in the article. Further inquiries can be directed to the corresponding author.

## Supplementary Materials

The following supporting information can be downloaded at https://www.mdpi.com/article/10.3390/diagnostics16091252/s1.
