# Supplementary material for: Association Between Familial Mediterranean Fever and P-Wave Dispersion Under Colchicine Treatment
Source: Diagnostics (Basel). 2026 Apr 22;16(9):1252. doi: 10.3390/diagnostics16091252 (PMC13162951; doi:10.3390/diagnostics16091252)
Supplement: Supplementary file 1 [file diagnostics-16-01252-s001.zip › diagnostics-4229794-supplementary.pdf]

Supplementary Material

JBI Critical Appraisal — Analytical Cross-Sectional Study

Tool Reference: Barker TH, Hasanoff S, Aromataris E, Stone JC, Leonardi-Bee J, Sears K, et al. The revised JBI critical appraisal tool for the assessment of risk of bias for analytical cross-sectional studies. JBI Evid Synth. 2026;24(3):401-8.

Study: Association Between Familial Mediterranean Fever and P-Wave Dispersion Under Colchicine Treatment

Cure O, Durak H, Çetin M, Kızılkaya B | Recep Tayyip Erdoğan University Faculty of Medicine, Rize, Türkiye

No.	Appraisal Criterion	Judgment	Justification Based on Study Data
1	Were the criteria for inclusion in the sample clearly defined?	Yes	Inclusion and exclusion criteria are explicitly stated (Tel-Hashomer criteria for FMF diagnosis, colchicine ≥1 mg/day for ≥1 year, age/sex-matched controls). Patients with structural heart disease, severe electrolyte disturbances, or antiarrhythmic drug use were excluded.
2	Were the study subjects and the setting described in detail?	Yes	The study was conducted at Recep Tayyip Erdogan University Faculty of Medicine, Rize, Turkey. All participants were of Turkish origin. 97 FMF(+) and 97 age- and sex-matched FMF(−) individuals were included. Clinical, laboratory, and demographic characteristics are described in Table 1.

No.	Appraisal Criterion	Judgment	Justification Based on Study Data
3	Was the exposure measured in a valid and reliable way?	Yes	FMF status (the primary exposure) was confirmed using validated Tel-Hashomer criteria. Colchicine dose was recorded in mg/day from electronic medical records. The attack-free period was defined using standardized clinical criteria (absence of typical FMF symptoms for ≥2 weeks prior to enrollment).
4	Were objective, standard criteria used for measurement of the condition?	Yes	P-wave dispersion was measured on standard 12-lead ECGs obtained at 25 mm/s and 10 mm/mV by two independent cardiologists blinded to clinical data. Reproducibility was assessed with ICC: inter-observer ICC = 0.82, intra-observer ICC = 0.91, indicating good to excellent agreement.
5	Were confounding factors identified?	Yes	Potential confounders including cardiovascular comorbidities (hypertension, hyperlipidemia, CAD, DM), medications known to affect ECG parameters (calcium channel blockers, beta-blockers, ACE inhibitors/ARBs, statins), and laboratory variables (WBC, CRP, HDL-C) were identified a priori and included in multivariable regression analyses.

No.	Appraisal Criterion	Judgment	Justification Based on Study Data
**6**	Were strategies to deal with confounding factors stated?	**Yes**	Age- and sex-matching using the nearest neighbour method was performed to control for these key confounders. Additionally, multivariable backward linear regression analyses were conducted to identify independent predictors of P-wave dispersion while adjusting for clinical and laboratory confounders. Two separate models were run to address multicollinearity between WBC and neutrophil counts.
**7**	Were the outcomes measured in a valid and reliable way?	**Yes**	The primary outcome (P-wave dispersion) was measured using a standardized protocol on magnified digital ECG images. Two blinded cardiologists performed measurements independently. Reproducibility was confirmed via ICC analysis (inter-observer 0.82; intra-observer 0.91). Validated statistical methods (Mann-Whitney U, Spearman correlation, multivariable regression) were used.

No.	Appraisal Criterion	Judgment	Justification Based on Study Data
**8**	Was appropriate statistical analysis used?	**Yes**	Normality was assessed with the Shapiro-Wilk test. Appropriate tests were selected accordingly (t-test or Mann-Whitney U; Pearson or Spearman correlation). Multivariable backward linear regression was used to identify independent predictors. Post-hoc power analysis confirmed adequate study power (0.92 at $\alpha=0.05$). Collinearity was addressed by running separate regression models. SPSS v29 was used for all analyses.

Overall Appraisal: All 8 JBI criteria were judged as "Yes". The study demonstrates adequate methodological quality with clearly defined inclusion criteria, validated exposure and outcome measurements, appropriate confounder identification and control, and robust statistical analyses. The findings are considered suitable for inclusion in further evidence synthesis.

Response Options: Yes / No / Unclear / Not Applicable
